# White matter hyperintensities in Burning Mouth Syndrome assessed according to the Age-Related White Matter Changes scale

**DOI:** 10.3389/fnagi.2022.923720

**Published:** 2022-09-01

**Authors:** Daniela Adamo, Federica Canfora, Elena Calabria, Noemi Coppola, Stefania Leuci, Giuseppe Pecoraro, Renato Cuocolo, Lorenzo Ugga, Luca D’Aniello, Massimo Aria, Michele D. Mignogna

**Affiliations:** ^1^Department of Neuroscience, Reproductive Sciences and Dentistry, University of Naples Federico II, Naples, Italy; ^2^Department of Clinical Medicine and Surgery, University of Naples Federico II, Naples, Italy; ^3^Department of Advanced Biomedical Sciences, University of Naples Federico II, Naples, Italy; ^4^Department of Social Sciences, University of Naples Federico II, Naples, Italy; ^5^Department of Economics and Statistics, University of Naples Federico II, Naples, Italy

**Keywords:** Burning Mouth Syndrome, white matter hyperintensities, Age-Related White Matter Changes, dementia, pain

## Abstract

**Background:**

White matter hyperintensities (WMHs) of the brain are observed in normal aging, in various subtypes of dementia and in chronic pain, playing a crucial role in pain processing. The aim of the study has been to assess the WMHs in Burning Mouth Syndrome (BMS) patients by means of the Age-Related White Matter Changes scale (ARWMCs) and to analyze their predictors.

**Methods:**

One hundred BMS patients were prospectively recruited and underwent magnetic resonance imaging (MRI) of the brain. Their ARWMCs scores were compared with those of an equal number of healthy subjects matched for age and sex. Intensity and quality of pain, psychological profile, and blood biomarkers of BMS patients were further investigated to find potential predictors of WMHs. Specifically, the Numeric Rating Scale (NRS), Short-Form McGill Pain Questionnaire (SF-MPQ), Hamilton rating scale for Depression and Anxiety (HAM-D and HAM-A), Pittsburgh Sleep Quality Index (PSQI), Epworth Sleepiness Scale (ESS) were administered.

**Results:**

The BMS patients presented statistically significant higher scores on the ARWMCs compared to the controls, especially in the right frontal, left frontal, right parietal-occipital, left parietal-occipital, right temporal and left temporal lobes (*p*-values: <0.001, <0.001, 0.005, 0.002, 0.009, 0.002, and <0.001, respectively). Age, a lower educational level, unemployment, essential hypertension, and hypercholesterolemia were correlated to a higher total score on the ARWMCs (*p*-values: <0.001, 0.016, 0.014, 0.001, and 0.039, respectively). No correlation was found with the blood biomarkers, NRS, SF-MPQ, HAM-A, HAM-D, PSQI, and ESS.

**Conclusion:**

Patients with BMS showed a higher frequency of WMHs of the brain as suggested by the higher ARWCs scores compared with the normal aging of the healthy subjects. These findings could have a role in the pathophysiology of the disease and potentially affect and enhance pain perception.

## Introduction

White matter hyperintensities (WMHs) are the most frequent macrostructural brain changes occurring in later life, showing a prevalence rate ranging between 39% and 96% (Debette et al., 2019). Although initially considered a normal, age-related finding, recent investigations have proved that WMHs of the brain must be considered as neuroimaging markers of brain frailty (Frey et al., 2019).

The pathological changes of WMHs are related to the white matter damage localization, which is characterized by a partial loss of myelin and axons, a loss of glial cells, a dilatation of the perivascular spaces, and fibro-hyalinotic vessel changes (Brown et al., 2018; Debette et al., 2019). WMHs appear on magnetic resonance imaging (MRI) as hypointensities on T1-weighted imaging and hyperintensities on T2-weighted imaging and FLAIR (fluid-attenuated inversion recovery sequences) (Griffanti et al., 2018). Although automated volumetric quantification based on MRI is the ideal method to quantify the grade and severity of WMHs, this technique is restricted to research purposes (Frey et al., 2019; Bauer et al., 2021). On the other hand, the use of visual rating scales, such as the Age-Related White Matter Changes scale (ARWMCs), is more applicable and commonly used as a qualitative tool in daily practice for the assessment of WMHs. The ARWMCs is simple to use and has shown an excellent inter rater reliability in several studies (Wahlund et al., 2001; Xiong et al., 2010).

Little is known about the natural history of WMHs, either in terms of their onset or their progression, but aging and cardiovascular risk factors, particularly diastolic hypertension, are considered the most prominent risk factors for WMH development (Chen et al., 2021).

Several longitudinal studies have shown that WMHs are a predictor of the future risk of stroke, cognitive decline, dementia, depression, disability and mortality in the general population (Jokinen et al., 2005). In addition, recent meta-analysis studies have demonstrated that WMHs increase three-fold the risk of dementia and stroke and double the risk of death (Debette et al., 2019).

Moreover, WMHs and lacunes are commonly seen on MRI scans of the brain in patients with chronic pain conditions (Aradi et al., 2013; Higgins et al., 2018). These lesions may increase the risk of cognitive decline and dementia, accelerating aging and thus contributing to disability (Korczyn et al., 2012; Murman, 2015). The role of WMHs in patients with chronic pain is still debated (Buckalew et al., 2013; Binnekade et al., 2019). However, they may potentially reflect macrostructural white matter damage, also influencing interconnections among multiple regions of the brain, mainly from the cortical to subcortical areas, which in turn may affect the descending pathway of the modulatory system of pain, playing a significant role in the amplification and processing of the pain (Wozniak and Lim, 2006; Kim et al., 2022).

Burning Mouth Syndrome (BMS) is a type of complex chronic neuropathic orofacial pain disorder characterized by a generalized or localized intraoral burning or dysesthetic sensation or pain of the oral mucosa without any evidence of any specific mucosal lesions and/or laboratory findings [International Classification of Orofacial Pain, 1st edition (ICOP) Cephalalgia, 2020]. The oral burning is usually bilateral but sometimes also unilateral, following the distributions of one or more branches of the trigeminal nerves. Notably, most of the patients report subjective xerostomia, dysgeusia, a bitter/metallic taste, sialorrhea, an intraoral foreign body sensation, itching, a tingling sensation and globus as additional symptoms (Moisset et al., 2016). The worldwide prevalence of the disease is around 4% but this varies considerably in relation to the different definitions of BMS adopted with consequent different inclusion criteria considered [Headache Classification Committee of the International Headache Society (IHS) The International Classification of Headache Disorders, 3rd edition, Cephalalgia, 2018]. The prevalence increases in post-menopausal women (18%), with a female-to-male ratio of 3:1 (Coculescu et al., 2014). The aetiopathogenesis is still debated but it is considered to be multifactorial in which the peripheral small fibers neuropathy is associated with the central nervous dysfunction (Lopez-Jornet et al., 2017). Indeed, neuroimaging studies have reported structural brain changes similar to those observed in neurodegenerative diseases such as Alzheimer’s disease (AD) and vascular dementia (VaD), suggesting a bidirectional correlation between dementia and chronic orofacial pain (COFP) (Corbett et al., 2014; Innes and Sambamoorthi, 2020). It is known that patients with cognitive decline report a high level of pain (Higgins et al., 2018) and, in turn, any persistent and untreated pain may accelerate memory decline and increase the probability of dementia (Corbett et al., 2014; Innes and Sambamoorthi, 2020). In this context, WMHs could be considered to have a role in the onset and exacerbation of both conditions and the quantity and distribution of the WMHs may be a specific target in terms of differentiating the diseases (Debette et al., 2019; Bauer et al., 2021).

Therefore, investigating how these structural brain changes impact on brain function and affect cognitive skills and pain processing is crucial for the management of the rapidly aging population (Stephen et al., 2021; Chowdhary et al., 2022). Indeed, until now, the therapeutic approach to the dementia subtypes remains limited. Therefore, identifying and treating remediable risk factors and predictors, such as WMHs and chronic pain conditions, in addition to the control of cardiovascular illnesses, may contribute to the prevention of these disabling neurodegenerative diseases (Corbett et al., 2014; Innes and Sambamoorthi, 2020).

In one of our previous studies, we have demonstrated for the first time that patients with BMS suffer from cognitive decline mainly in attention, working memory and executive functions, showing a higher score on the ARWMCs in the temporal lobes compared with healthy subjects, thereby suggesting a higher percentage of WMHs in this area of the brain in patients affected by BMS (Canfora et al., 2021).

Consequently, we have now performed another study on a wide sample of patients with BMS in order to confirm our previous findings. Our specific hypothesis has been that patients with BMS could show a higher prevalence of WMHs compared with pain-free controls of the same age, resulting in the premature aging of the brain, where WMHs work as pathogenic contributors together with the other well-known risk factors (Livingston et al., 2020). Therefore, the primary end point of the present study has been to assess the prevalence and extent of WMHs of the brain by means of the ARWMCs in a wide sample of BMS patients, compared with a control group of healthy subjects matched for gender, age and educational level, also exploring differences in global and regional WMH scores in the patients at different ages. Our second objective has been to identify the potential predictors of WMHs in patients with BMS, taking into account the sociodemographic profile (age, gender, education, employment and marital status), body mass index (BMI), risk factors (smoking and alcohol use), disease onset, sleep duration, other systemic comorbidities, drug intake, blood biomarkers, pain evaluation, symptomatology and psychological factors (Debette et al., 2019; Walsh et al., 2020, 2021).

## Methods

### Study design and participants

A case-control study was conducted on patients examined between November 2019 and December 2020 at the University of Naples “Federico II” Oral Medicine unit with the approval the Ethical Committee of the University (Approval Number: 251/19- date of approval 20th February 2019). The study was carried out in accordance with the ethical principles of the World Medical Association Declaration of Helsinki and the methodology conformed with the Strengthening the Reporting of Observational Studies in Epidemiology (STROBE) guidelines for observational studies (von Elm et al., 2014).

**FIGURE 1:**
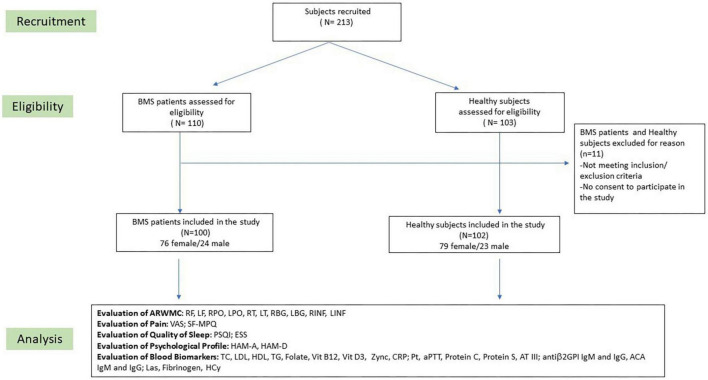
Flow chart of the study. BMS, Burning Mouth Syndrome; ARWMC, Age Related White Matter Changes; RF, right frontal; LF, left frontal; RPO, right parieto-occipital; LPO, left parieto-occipital; RT, right temporal; LT, left temporal; RBG, right basal ganglia; LBG, left basal ganglia; RINF, right infratentorial; LINF, left infratentorial; VAS, Visual Analog Scale; SF-MPQ, Short-Form McGill Pain Questionnaire; PSQI, Pittsburgh Sleep Quality Index; ESS, Epworth Sleepiness Scale; HAM-A, Hamilton Anxiety Rating Scale; HAM-D, Hamilton Depression Rating Scale; TC, total cholesterol; LDL, low-density lipoprotein cholesterol; HDL, high-density lipoprotein cholesterol; TG, triglycerides; CRP, C-reactive protein; PT, prothrombin time; aPTT, partial thromboplastin time; AT III, plasma antithrombin III; Antiβ2GPI, anti-β2-glycoprotein I antibodies; ACA, anti-cardiolipin antibodies; Las, lupus anticoagulants; Hcy, homocysteine.

Potentially eligible participants were consecutively invited to participate in the study and their written informed consent was obtained. The recruitment planning and development has been described in the flow-chart of the study (Figure 1). Patients with a confirmed diagnosis of BMS based on the International Classification of Orofacial Pain, 1st edition [International Classification of Orofacial Pain, 1st edition (ICOP) Cephalalgia, 2020] were included in accordance with the following inclusion criteria: (i) patients of any race or gender; (ii) a complaint of oral burning recurring daily for >2 h per day for >3 months; (iii) the absence of any clinical mucosal alterations on inspection of the oral cavity; and (iv) normal blood test findings (including blood count, blood glucose levels, glycated hemoglobin, serum iron, ferritin and transferrin). On the contrary, patients were excluded in accordance with the following exclusion criteria: (i) the presence of any disease that could be recognized as a causative factor of BMS, (ii) a history of a psychiatric disorder or a neurological or organic brain disorder, (iii) a history of alcohol or substance abuse, (iv) ongoing treatment with systemic drugs possibly associated with oral symptoms; (v) ongoing treatment with psychotropic drugs; (vi) a MRI scan performed after 4 weeks of the examination; and (vii) the presence of Obstructive Sleep Apnea Syndrome (OSAS).

After the enrollment of the BMS patients, subjects who had undergone an MRI examination as a healthy control for a previous study were recruited and matched according to the age and gender of the case group (Cocozza et al., 2017).

### Clinical assessment

All the BMS patients underwent a thorough medical assessment, consisting in a general examination, an intra- and extra-oral clinical examination, a psychiatric evaluation, and MRI scans. At admission, venous blood samples were collected in the morning for all the patients and relevant clinical data were recorded in accordance with a predefined form. The following socio-demographic and medical information was collected: sex, age, years of education, family situation (single, married, divorced or widowed), employment (employed, unemployed or retired), BMI (calculated as weight in kilograms divided by height in meters squared) (Peterson et al., 2016), disease onset (in years), sleep duration (in hours), risk factors (current smoking status, alcohol consumption and lack of physical activity), systemic diseases and drug consumption.

To standardize the clinical procedures, the oral examination was performed by an oral medicine specialist (DA) while the psychiatric assessment was performed by a psychiatrist (GP), both having more than 10 years of experience in the psychiatric and pain assessment of elderly subjects with chronic orofacial pain. The oral symptomatology was assessed in detail, and data were collected on the type of the oral symptoms (pain, burning sensation, xerostomia, dysgeusia, sialorrhea, globus pharyngeus, itching, intraoral foreign body sensation, occlusal dysesthesia, change in tongue morphology, oral dyskinesia or dysosmia), on the oral localization of the pain or burning sensation, on the worst symptom reported and on the diurnal patterns of the symptoms. Additionally, the patients were asked about the timing of the disease onset, any suspected cause triggering the oral symptoms, the number and type of specialists consulted prior to the diagnosis of BMS, any previous personal or family history of mood disorders and the presence and onset of insomnia.

### Pain, psychological assessment and quality of sleep assessment

A predefined set of questionnaires, all validated in Italian, was administered to the BMS patients included in the study. The intensity and quality of pain were evaluated with the Numeric Rating Scale (NRS) and the Short-form McGill Pain Questionnaire (SF-MPQ), respectively. The NRS score ranges from 0 to 10 (0 = no oral symptoms and 10 = the worst imaginable discomfort) (Hawker et al., 2011). The SF-MPQ measures the sensory, affective and evaluative aspects of the perceived pain (Melzack, 1987; Hawker et al., 2011) and consists of 15 items each scored from 0 (none) to 3 (severe), with a total score given by the sum of all the item scores (range 0–45). There are no established thresholds of pain severity, with higher scores indicating worse pain. The psychological profile was explored by using the Hamilton rating scale for Depression (HAM-D) (Hamilton, 1960, 1967) and the Hamilton rating scale for Anxiety (HAM-A) (Hamilton, 1959), both tools being clinician-administered assessment scales. The HAM-D consists of 21 items and the total scores can range from 0 to 54. Scores greater than 7 indicate the presence of depression, specifically between 7 and 17 of mild depression, between 18 and 24 of moderate depression, and over 24 of severe depression (Morriss et al., 2008). The HAM-A consists of 14 items and measures both psychic anxiety and somatic anxiety and the total score ranges from 0 to 56. Scores above 17 indicate mild anxiety, 18–24 moderate anxiety and over 25 severe anxiety (Hamilton, 1967).

Any sleep disorders were assessed by investigating the subjective sleep quality and daytime sleepiness through the Pittsburgh Sleep Quality Index (PSQI) and Epworth Sleepiness Scale (ESS), respectively, both consisting of self-rated questionnaires. The PSQI (Buysse et al., 1989) explores the quality of sleep over a 1-month time interval generating seven “component” scores (0–3): subjective sleep quality, sleep latency, sleep duration, habitual sleep efficiency, sleep disturbances, use of sleeping medication and daytime dysfunction (Carpenter and Andrykowski, 1998). The total score is obtained by the sum of all the sub-scores and ranges between 0 and 21. A total score greater than five discriminates poor sleepers from good sleepers with a high sensitivity (90%–99%) and specificity (84%–87%) (Curcio et al., 2013). The ESS (Johns, 1991) evaluates the subject’s general level of daytime sleepiness and is characterized by eight items assessing the propensity of falling asleep in eight common situations with scores ranging from 0 (no chance of dozing) to 3 (a high chance of dozing). The ESS total score ranges from 0 to 24, with a cut-off value >10 indicating excessive daytime sleepiness (Vignatelli et al., 2003).

### Blood sampling, laboratory tests and biochemical markers

A blood test was performed to evaluate the general health status and levels of blood lipids [total cholesterol (TC), low-density lipoprotein cholesterol (LDL), high-density lipoprotein cholesterol (HDL), triglycerides (TG)]; deficiency [Folate levels, Vitamin B12, Vitamin D3, serum Zinc] and C-reactive protein (CRP) levels. This study also included a thrombophilia screening [prothrombin time (PT), partial thromboplastin time (aPTT), protein C and protein S activities, plasma antithrombin III (AT III), anti-β2-glycoprotein I antibodies (antiβ2GPI IgM and IgG), anti-cardiolipin antibodies (ACA IgM and IgG), lupus anticoagulants (Las), fibrinogen levels and homocysteine serum levels (Hcy)].

### Magnetic resonance imaging acquisition and white matter hyperintensity assessment

Within 4 weeks of the baseline examination, all the BMS subjects included underwent magnetic resonance imaging (MRI) of the brain using a standard protocol.

The MRI was performed on a 1.5-T scanner (Gyroscan Intera, Philips Medical Systems, Amsterdam, Netherlands) using a protocol which included a fluid-attenuated inversion recovery sequence (TR: 8005 ms, TE: 100 ms, TI: 2200 ms, matrix: 256 × 192, slice thickness: 5 mm) and a turbo spin-echo T2-weighted sequence (TR: 4400 ms, TE: 100 ms, matrix: 256 × 192, slice thickness: 5 mm).

Two neuroradiologists (LU and RC), blind to the clinical data, rated the WMH severity for the participants in the study using the ARWMCs score. Any discrepancies were solved by consensus.

In accordance with the original study of the ARWMCs (Wahlund et al., 2001), we defined WMHs as ill-defined hyperintensities ≥5 mm on both T2-weighted and FLAIR images. This scale grades the severity of WMHs in five brain regions: the frontal lobe, parieto-occipital region, temporal lobe, infratentorial region, and basal ganglia on a four-point scale (score 0 = no lesions, 1 = focal lesions; 2 = the beginning of a confluence of lesions and 3 = a diffuse involvement). The results can be presented as a total score or global score (Wahlund et al., 2001). In this study, the total score was used, representing the sum of the scores for each region in both hemispheres, a total which ranges from 0 to 30.

### Outcomes of the study

The primary outcome of the study was to evaluate the prevalence of WMHs assessed by the ARWMCs in the brain of BMS patients and healthy subjects in order to highlight any potential differences in terms both of the ARWMC total score and the sub-scores between the case group and control group. The secondary outcome was to identify any potential predictor of WMHs and higher ARWMCs scores in BMS patients by considering sociodemographic variables, clinical variables, pain and psychological factors.

In this paragraph, we report the list of variables investigated for this purpose. For analytical reasons, the quantitative variables and qualitative variables were distinguished based on the type of data, continuous for the former and categorical for the latter. The quantitative variables were: age (in years), education (in years), BMI, pain (NRS, SF-MPQ), sleep duration in hours (VAS, SF-MPQ), depression (HAM-D), anxiety (HAM-A), quality of sleep (PSQI, ESS), serological levels of folate, vitamin B12, vitamin D3, PT, aPTT, INR, protein C, protein S, AT III, Anti-β2GPI IgG, Anti-β2GPI IgM, ACA IgG, ACA IgM, Las, fibrinogen, zinc, homocysteine, CRP, number of systemic comorbidities total drug intake, number of oral symptoms and disease onset in years. The qualitative variables were: gender (male/female), marital status (married/not married), employment status (employed/not employed), smoking status (smoker/non-smoker), alcohol assumption (yes/no), essential hypertension (yes/no), hypercholesterolemia (yes/no); hyperhomocysteinemia (HHCys) (yes/no), dysgeusia (yes/no), xerostomia (yes/no), change in tongue morphology (yes/no), and globus pharyngeus (yes/no).

Specifically:

-the patient was considered to suffer from essential hypertension if she/he had blood pressure (BP) higher than 140/90 mm Hg (SBP/DBP), according to the current ACCF/AHA criteria for uncomplicated hypertension in the elderly (Messerli et al., 2011) or if she/he was taking antihypertensives;

-the patient was considered to suffer from hypercholesterolemia using the cut-offs for the Metabolic Syndrome recommended by the NCEP- ATPIII (Welty, 2001): CT ≥ 200 mg/dl; or if she/he was in treatment with statins.

-the patient was considered to suffer from HHCys using the cut-off value >11 μMOL/L (Ansari et al., 2014).

### Statistical analysis

The patients and controls were matched by age and gender. First, we recruited the patients and calculated their gender distribution and average age; then, we selected the controls to obtain a matched sample.

The sample size, equal to 100 BMS patients and 102 controls, was set by fixing a power test value (1-Beta) at no less than 99%, associated with a significance of no more than 5%. This sample size was calculated using the effect size value of 0.69, measured in a previously published research study regarding the ARWMCs (Canfora et al., 2021). The calculations were computed using the Gpower software (v 3.1.9). Descriptive statistics, including means and standard deviations for the normally distributed data, and medians and interquartile ranges (IQR) for the non-normally distributed data, were used to analyze the sociodemographic and clinical characteristics, pain measures (NRS, T-PRI) and psychological factors (HAM-A, HAM-D, PSQI, ESS) of the BMS patients. Any significant differences in the median ARWMCs total scores and sub-scores between the BMS patients and healthy subjects were evaluated by using the Mann Whitney test. Any comparisons of the median ARWMCs total scores and sub-scores were also performed by stratifying the BMS patients and controls depending on the specific age group, namely a first group of participants aged under 65, a second group aged 65–69, a third group aged 70–74 and a fourth group aged over 75. *P*-values less than 0.05 or 0.01 were considered moderately or strongly significant, respectively. The Spearman’s correlation was computed to analyze the correlation between the above quantitative and qualitative predictors and the ARWMCs total scores. Any qualitative and quantitative variables resulting statistically correlated to the ARWMCs total scores were further correlated to the sub-scores of the ARWMCs (right and left frontal lobes, right and left parieto-occipital lobes, right and left temporal lobes, right and left basal ganglia and right and left infratemporal lobes scores). For the latter analysis, the significance difference between the correlation coefficients was measured using the Bonferroni correction. The test was considered significant with a *P*-value < 0.005. All the statistical analyses were performed using the SPSS software v. 26.

## Results

The sociodemographic profiles, risk factors, systemic diseases, drug consumption and biochemical blood biomarkers are shown in Table 1 (Supplementary material 1).

**TABLE 1:** Sociodemographic profile, risk factors and analysis of biochemical blood markers in 100 BMS patients.

Demographic variables	BMS patients
**Gender** Male Female	**Frequency (%)** 24 (24) 76 (76)
**Age** (in years)	**Mean ± SD** 65.34 ± 8.14
**Education** (in years)	**Mean ± SD** 9.09 ± 4.44
**Family situation** Single Married Divorced Widowed	**Frequency (%)** 6 (6) 82 (82) 3 (3) 9 (9)
**Employment** Employed Unemployed Retired	**Frequency (%)** 17 (17) 46 (46) 37 (37)
**Body mass index**	**Mean ± SD** 27.11 ± 3.99

**Risk factors**	**Frequency (%)**

**Smoking** Never <5 cigarettes 5–10 cigarettes 10–15 cigarettes >15 cigarettes	76 (76) 7 (7) 4 (4) 7 (7) 6 (6)
**Alcohol use** Never Yes (1–2 units/week) Yes (2–3) Yes (>3)	85 (80) 10 (10) 5 (5) 0 (0)

**Biochemical blood markers**	**(Median; IQR)**

TC (mg/dl)	200 [174.25–220]
LDL (mg/dl)	126 [105–143.25]
HDL (mg/dl)	55 [45–65]
TG (md/dl)	120 [94–170]
Folate (ng/ml)	6.35 [4.07–9.22]
Vitamin B12 (pg/dl)	348.5 [269.25–438.5]
Vitamin D3 (ng/ml)	25.1 [19.82–36.23]
PT (s)	11.3 [11–12.15]
INR	0.99 [0.94–1.05]
aPTT (s)	28.7 [25.9–31.1]
PROTEIN C (%)	113.4 [103–124]
PROTEIN S (%)	93 [80.5–101.2]
AT III (%)	101.6 [90.88–110]
Anti-β2GPI IgG (U/ml)	1.4 [1.3–2.2]
Anti-β2GPI IgM (U/ml)	1.3 [1–1.4]
ACA IgG (U/ml)	2 [1.5–3]
ACA IgM (U/ml)	1.9 [1.1–2.4]
Las (RATIO)	1.1 [1.1–1.1]
Fibrinogen (mg/dl)	320 [284.25–366]
Homocysteine (μ/MOL/L)	13.95 [10.95–16]
Zinc (μg/L)	730 [720–812.5]
CRP (U/ml)	1.1 [0.39–1.3]

BMS, Burning Mouth Syndrome; TC, total cholesterol; LDL, low-density lipoprotein; HDL, high-density lipoprotein; TG, triglycerides; PT, prothrombin time; INR, international normalized ratio; aPTT, partial thromboplastin time; AT III, antithrombin 3; Anti-B2GPI, anti-B2-glycoprotein 1; ACA, anticardiolipin antibodies; LAs, lupus anticoagulants; CRP, C reactive protein.

**FIGURE 2:**
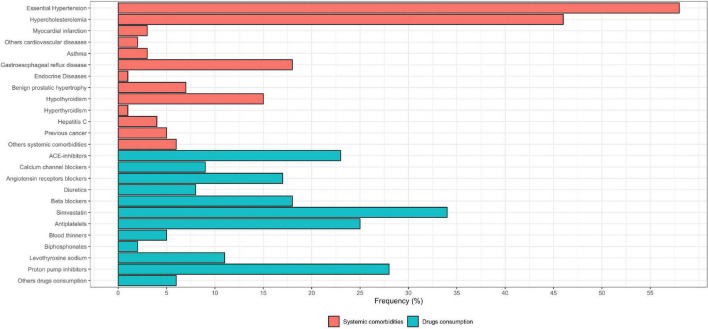
Boxplot of systemic comorbidity and drug intake in BMS patients.

Figure 2 shows the systemic comorbidity and drug intake in BMS patients (Supplementary material 1). Table 2 shows the prevalence of the oral symptoms, the location, timing and pattern of the pain, the number of specialists consulted and typology of referrals, the analysis of the intensity and quality of pain, and the analysis of the psychological profile of the patients (Supplementary material 1).

**TABLE 2:** Prevalence of oral symptoms, location, timing and pattern of pain and worst symptom, disease onset, number of doctors and typology of referrals, cause of disease and analysis of pain, psychological profile and quality of sleep in BMS patients.

Oral symptoms	Frequency (%)
Burning	100 (100)
Xerostomia	56 (56)
Dysgeusia	44 (44)
Change in tongue morphology	44 (44)
Globus pharyngeus	37 (37)
Intraoral foreign body sensation	21 (21)
Sialorrhea	16 (16)
Occlusal dysesthesia	13 (13)
Tingling sensation	10 (10)
Itching	9 (9)
Oral dyskinesia	3 (3)
Dysosmia	3 (3)

**Location of pain/burning**	**Frequency (%)**

Tongue	94 (94)
Lips	64 (64)
Anterior palate	60 (60)
Gums	55 (55)
Cheeks	53 (53)
Floor of the mouth	44 (44)
Soft palate	41 (41)

**Worst symptom**	**Frequency (%)**

Burning Change in tongue morphology Dysgeusia Xerostomia Globus Sialorrhea Intraoral foreign body sensation	74 (74) 12 (12) 6 (6) 4 (4) 2 (2) 1 (1) 1 (1)

**Diurnal pattern of symptoms**	**Frequency (%)**

Same morning/afternoon/evening Worst in the afternoon/evening Worst in the morning Continuous Intermittent	50 (50%) 46 (46%) 4 (4%) 69 (69%) 31 (31%)

**Disease onset (months)**	**Mean ± SD**

	29 ± 47.67

**Number of doctors consulted prior to diagnosis of BMS**	**Mean ± SD**

	2.6 ± 1.56

**Referrals**	**Frequency (%)**

Dentist Physician Maxillofacial surgeon Gastroenterologist Otolaryngologist Dermatologist Neurologist Psychiatrist	90 (90) 53 (53) 17 (17) 17 (17) 15 (15) 6 (6) 8 (8) 8 (8)

**Cause of disease attributed by patient**	**Frequency (%)**

Dental treatment Stressful life event Not attributed	22 (22) 12 (12) 66 (66)

**Pain**	**Median; IQR**

**NRS**	10 [9.75–10]
**SF-MPQ**	9 [5.75–13]

**Psychological profile**	**Median; IQR**

**HAM-A**	18 [15–21]
**HAM-D**	18 [14–21]

**History of previous mood disorders**	**Frequency (%)**

	30 (30)

**Familiarity of mood disorders**	**Frequency (%)**

	4 (4)

**Sleep**	**Median; IQR**

**Sleep duration (in hours)**	5 [5–6]
**Insomnia onset (in years)**	4 [2–6]
**PSQI**	8 [8–10]
**ESS**	5 [4–7]

**Onset of insomnia prior to BMS**	**Frequency (%)**

	74 (74)

BMS, Burning Mouth Syndrome; NRS, Numeric Rating Scale; SF-MPQ: Short-Form McGill Pain Questionnaire; HAM-A, Hamilton anxiety; HAM-D, Hamilton depression; PSQI, Pittsburgh Sleep Quality Index; ESS, Epworth Sleepiness Scale.

**FIGURE 3:**
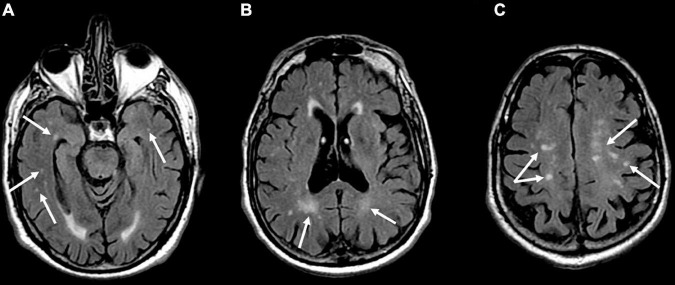
Axial T2-weighted fluid-attenuated inversion recovery (FLAIR) images show multiple hyperintense signal of gliotic foci in the temporal **(A)**, parietal **(B)**, and frontal **(C)** white matter in a 60-year-old male patient affected by Burning Mouth Syndrome. ARWMC total score: 8; RF: 1; LF: 1; RPO: 2; LPO: 2; RT: 1; LF: 1.

The analysis of the ARWMCs scores of the BMS patients compared with the healthy subjects is summarized in Table 3. WMHs were identified in 50% of the patients with BMS and in 26% of the healthy subjects matched for age and sex. There was a statistically significant difference in the total scores of the ARWMCs between the patients and controls (*p*-value: <0.001^**^). Specifically, there were significant differences in the scores of the right-frontal (RF) lobe and left-frontal (LF) lobe (*p*-values: <0.001^**^), of the right-parieto-occipital (RPO) lobe and left-parieto-occipital (LPO) lobe (*p*-values: 0.005^**^ and 0.002^**^, respectively), and of the right-temporal (RT) lobe and left-temporal (LT) lobe (*p*-values: 0.009^**^ and 0.002^**^, respectively) (Figure 3). No statistically significant differences were detected in the right-basal ganglia (RBG), left-basal-ganglia (LBG), right-infratentorial (RINF) region and left-infratentorial (LINF) region between the cases and controls. Overall, these findings demonstrate the higher prevalence of WMHs in the frontal, parieto-occipital and temporal areas in patients with BMS in comparison with the control group without BMS. In particular, the mean of the ARWMCs total score in the BMS patients was 2.99 while in the controls it was 1.42.

**TABLE 3:** Analysis of the ARWMC scores of the patients with BMS and the healthy controls.

ARWMC	BMS patients		Control group		
	Me [Q1–Q3]	Min.–Max.	Me [Q1–Q3]	Min.–Max.	*P*-value
Right frontal (RF)	1 [0–1]	0–3	0 [0–1]	0–1	<0.001**
Left frontal (LF)	1 [0–1]	0–3	0 [0–1]	0–1	<0.001**
Right parieto-occipital (RPO)	0 [0–1]	0–3	0 [0–0]	0–2	0.005**
Left parieto-occipital (LPO)	0 [0–1]	0–3	0 [0–0]	0–2	0.002**
Right temporal (RT)	0 [0–0]	0–2	0 [0–0]	0–1	0.009**
Left temporal (LT)	0 [0–0]	0–2	0 [0–0]	0–1	0.002**
Right basal ganglia (RBG)	0 [0–0]	0–1	0 [0–0]	0–0	0.154
Left basal ganglia (LBG)	0 [0–0]	0–1	0 [0–0]	0–0	0.317
Right infra-tentorial (RINF)	0 [0–0]	0–1	0 [0–0]	0–1	0.984
Left infra-tentorial (LINF)	0 [0–0]	0–1	0 [0—0]	0–1	0.307
Total score	2 [0–4]	0–14	1 [0–2.75]	0–8	<0.001**

IQR is the interquartile range. A significant difference between medians was measured by the Mann–Whitney test.

*Significant 0.01 < *p* ≤ 0.05. **Significant *p* ≤ 0.01.

BMS, Burning Mouth Syndrome; ARWMC, Age Related White Matter Changes; LF, left frontal; RF, right frontal; LPO, left parieto-occipital; RPO, right parieto-occipital; LT, left temporal; RT, right temporal; LBG, left basal ganglia; RBG, right basal ganglia; LINF, left infra-tentorial; RINF, right infra-tentorial.

**FIGURE 4:**
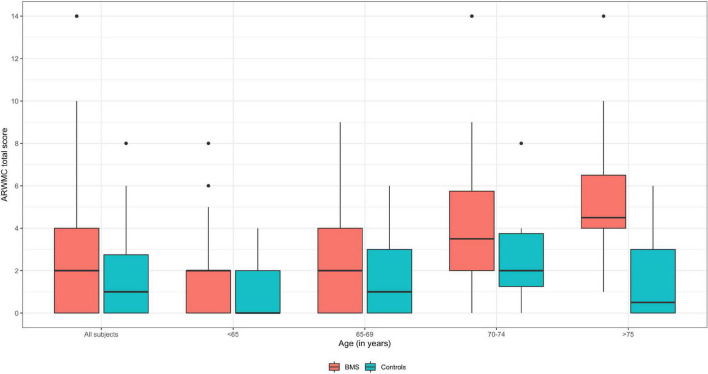
Boxplot of ARWMC total score of BMS and control subjects according to the age subgroups. Median values are highlighted by bold lines.

In detail, analyses of the ARWMCs scores were also computed comparing subgroups of patients and controls stratified according to specific age ranges (Table 4 and Figure 4). In the first subgroup of 41 BMS patients and 48 controls aged under 65 a statistically significant difference in the total ARWMCs score was found (*p*-value: 0.022*). Moreover, a strongly significant difference was found with respect to the score of the RF lobe (*p*-values: 0.009^**^) while the difference was moderately significant with regard to the LF lobe and LT lobe (*p*-values: 0.018* and 0.028* respectively). On the other hand, there was no statistically significant difference in the total ARWMCs score when comparing the 21 BMS patients and 28 healthy subjects aged 65–69 (*p*-value: 0.168). In this subgroup, however, a slightly significant difference was evident in the scores for the LF, RT and LT lobes (*p*-values: 0.047*, 0.044*and 0.044*, respectively). Interestingly, no differences were found either in the total ARWMCs score or in the sub-scores in the group of 26 BMS patients and 14 controls aged 70–74 years (ARWMC total score *p*-value: 0.159). On the contrary, a strongly statistically significant difference in the total score of the ARWMCs was found between the 12 BMS patients and 12 healthy subjects aged over 75 years (*p*-value: 0.004^**^), particularly in relation to the RF and LF lobes (*p*-values: 0.001^**^ and 0.012*, respectively), and in relation to the RPO and LPO lobes (*p*-values: 0.001^**^ and 0.001*, respectively). Taken together these results show that, given the presence of a higher prevalence of WMHs in the BMS patients compared to the healthy subjects, this difference is predominant among the participants aged under 65 and over 75 years. However, despite the fact that with the older age, the percentage of WMHs naturally increased, no significant differences were found between the cases and controls in the age group between 65 to 74.

**TABLE 4:** Analysis of the ARWMC scores of the patients with BMS and the healthy controls according to the age subgroups.

ARWMC	BMS 41 subjects Age (in years) < 65 (Median; IQR)	Controls 48 subjects Age (in years) < 65 (Median; IQR)	*P*-value
**Right frontal (RF)**	1 [0–1]	0 [0–1]	0.009**
**Left frontal (LF)**	1 [0–1]	0 [0–1]	0.018*
**Right parieto-occipital (RPO)**	0 [0–0]	0 [0–0]	0.889
**Left parieto-occipital (LPO)**	0 [0–0]	0 [0–0]	0.889
**Right temporal (RT)**	0 [0–0]	0 [0–0]	0.289
**Left temporal (LT)**	0 [0–0]	0 [0–0]	0.028*
**Right basal ganglia (RBG)**	0 [0–0]	0 [0–0]	0.289
**Left basal ganglia (LBG)**	0 [0–0]	0 [0–0]	1.000
**Right infra-tentorial (RINF)**	0 [0–0]	0 [0–0]	0.128
**Left infra-tentorial (LINF)**	0 [0–0]	0 [0–0]	0.289
**Total score**	2 [0–2]	0 [0–2]	0.022*

**ARWMC**	**BMS 21 subjects** **Age (in years) 65–69**	**Controls 28 subjects** **Age (in years) 65–69**	***P*-value**

**Right frontal (RF)**	1 [0–1]	0 [0–1]	0.125
**Left frontal (LF)**	1 [0–1]	0 [0–1]	0.047*
**Right parieto-occipital (RPO)**	0 [0–1]	0 [0–0]	0.332
**Left parieto-occipital (LPO)**	0 [0–1]	0 [0–1]	0.652
**Right temporal (RT)**	0 [0–0]	0 [0–0]	0.044*
**Left temporal (LT)**	0 [0–0]	0 [0–0]	0.044*
**Right basal ganglia (RBG)**	0 [0–0]	0 [0–0]	1.000
**Left basal ganglia (LBG)**	0 [0–0]	0 [0–0]	1.000
**Right infra-tentorial (RINF)**	0 [0–0]	0 [0–0]	0.409
**Left infra-tentorial (LINF)**	0 [0–0]	0 [0–0]	1.000
**Total score**	2 [0–4]	1 [0–3]	0.168

**ARWMC**	**BMS 26 subjects** **Age (in years) 70–74**	**Controls 14 subjects** **Age (in years) 70–74**	***P*-value**

**Right frontal (RF)**	1 [0.25–1]	1 [0.25–1]	0.555
**Left frontal (LF)**	1 [1–1]	1 [1–1]	0.525
**Right parieto-occipital (RPO)**	1 [0–1]	0 [0–1]	0.326
**Left parieto-occipital (LPO)**	1 [0–1]	0 [0–0.75]	0.054
**Right temporal (RT)**	0 [0–0]	0 [0–0]	0.473
**Left temporal (LT)**	0 [0–0]	0 [0–0]	0.473
**Right basal ganglia (RBG)**	0 [0–0]	0 [0–0]	1.000
**Left basal ganglia (LBG)**	0 [0–0]	0 [0–0]	1.000
**Right infra-tentorial (RINF)**	0 [0–0]	0 [0–0]	0.496
**Left infra-tentorial (LINF)**	0 [0–0]	0 [0–0]	0.496
**Total score**	3.5 [2–5.75]	2 [1.25–3.75]	0.159

**ARWMC**	**BMS 12 subjects** **Age (in years) ≥ 75**	**Controls 12 subjects** **Age (in years) ≥ 75**	***P*-value**

**Right frontal (RF)**	1 [1–1.25]	0 [0–1]	0.001**
**Left frontal (LF)**	1 [1–1.25]	0.5 [0–1]	0.012*
**Right parieto-occipital (RPO)**	1 [1–1]	0 [0–0]	0.001**
**Left parieto-occipital (LPO)**	1 [1–1.25]	0 [0–0]	0.001**
**Right temporal (RT)**	0 [0–0.25]	0 [0–0]	0.285
**Left temporal (LT)**	0 [0–0.25]	0 [0–0]	0.285
**Right basal ganglia (RBG)**	0 [0–0]	0 [0–0]	0.359
**Left basal ganglia (LBG)**	0 [0–0]	0 [0–0]	0.359
**Right infra-tentorial (RINF)**	0 [0–0]	0 [0–0]	0.166
**Left infra-tentorial (LINF)**	0 [0–0]	0 [0–0]	1.000
**Total score**	4.5 [4–6.5]	0.5 [0–3]	0.004**

IQR is the interquartile range. A significant difference between medians was measured by the Mann–Whitney test.

*Significant 0.01 < *p* ≤ 0.05. **Significant *p* ≤ 0.01.

BMS, Burning Mouth Syndrome; ARWMC, Age Related White Matter Changes; LF, left frontal; RF, right frontal; LPO, left parieto-occipital; RPO, right parieto-occipital; LT, left temporal; RT, right temporal; LBG, left basal ganglia; RBG, right basal ganglia; LINF, left infra-tentorial; RINF, right infra-tentorial.

Correlation analyses between the total ARWMCs score and the specific quantitative and qualitative variables were computed in order to identify predictors of higher ARWMCs scores in BMS patients (Table 5). With respect to the quantitative variables, the total ARWMCs score was positively correlated to age, the number of systemic comorbidities and the amount of drug intake (*p*-values: <0.001^**^, 0.007^**^, and 0.026*, respectively) and was negatively correlated only with educational level (*p*-value: 0.016*). With regard to the quantitative variables, a strongly positive correlation was also found in relation to essential hypertension (*p*-value: <0.001^**^), whereas there was a moderate positive correlation with regard to the employment status and hypercholesterolemia (*p*-values: 0.014* and 0.039*, respectively). No correlation was found between the total ARWMCs score and the intensity and quality of the burning/pain and the more frequent additional symptoms. In addition, no correlation was found between the total ARWMCs score and anxiety, depression, sleep disturbance and disease onset.

**TABLE 5:** Linear correlation analysis between the total ARWMC score and the quantitative/qualitative predictors in BMS patients.

Predictors/ARWMC		Total score
Quantitative predictors		ρ (*P*-value)
**Age**		0.447 (<0.001**)
**Education level (in years)**		−0.24 (0.016*)
**BMI**		0.128 (0.205)
**NRS**		0.152 (0.131)
**HAM-A**		−0.017 (0.87)
**HAM-D**		0.003 (0.98)
**SF-MPQ**		0.058 (0.569)
**Sleep duration**		−0.045 (0.661)
**PSQI**		0.038 (0.711)
**ESS**		0.013 (0.9)
**Folate (ng/ml)**		−0.081 (0.42)
**Vitamin B12 (pg/dl)**		−0.003 (0.977)
**Vitamin D3 (ng/ml)**		−0.064 (0.528)
**PT (s)**		0.033 (0.745)
**INR**		−0.143 (0.156)
**aPTT (s)**		0.046 (0.647)
**PROTEIN C (%)**		0.076 (0.45)
**PROTEIN S (%)**		0.004 (0.97)
**AT III (%)**		−0.068 (0.499)
**Anti-β2GPI IgG (U/ml)**		−0.134 (0.182)
**Anti-β2GPI IgM (U/ml)**		0.026 (0.797)
**ACA IgG (U/ml)**		−0.145 (0.151)
**ACA IgM (U/ml)**		−0.095 (0.35)
**Las (RATIO)**		0.085 (0.404)
**Fibrinogen (mg/dl)**		0.022 (0.826)
**Zinc (μg/L)**		0.015 (0.879)
**Homocysteine (μ/MOL/L)**		0.181 (0.072)
**CRP (U/L)**		−0.133 (0.186)
**Number of systemic comorbidities**		0.266 (0.007**)
**Number of drug intake**		0.223 (0.026*)
**Number of oral symptoms**		0.119 (0.238)
**Disease onset**		−0.036 (0.720)

**Qualitative predictors**	**Median [Q1:Q3]**	***P*-value**

**Gender** *Female* *Male*	2 [0.75–4] 2 [0–4]	0.653
**Marital status** *Married* *Not married*	2 [0–4] 2 [1.25–5]	0.545
**Employment status** *Employed* *Not employed*	2 [0–2] 2 [1–4.5]	0.014*
**Smoking status** *Smoker* *Not smoker*	2 [0–4] 2 [1–4]	0.136
**Alcohol use** *Yes* *No*	3 [2–4] 2 [0–4]	0.430
**Essential hypertension** *Yes* *No*	3.5 [2–5] 1.5 [0–2]	<0.001**
**Hypercholesterolemia** *Yes* *No*	3 [2–5] 2 [0–4]	0.039*
**Hyperhomocysteinemia** *Yes* *No*	2 [0–5] 2 [0.5–4]	0.712
**Dysgeusia** *Yes* *No*	2 [0–4.25] 2 [1–4]	0.637
**Xerostomia** *Yes* *No*	2 [0–4] 3 [2–5]	0.061
**Change in tongue morphology** *Yes* *No*	2 [1–4] 2 [0–4]	0.597
**Globus** *Yes* *No*	2 [0–5] 2 [1–4]	0.519

*r* is Spearman’s correlation coefficient.

A significant difference between medians was measured by the Mann–Whitney test.

*Significant 0.01 < *p* ≤ 0.05. **Significant *p* ≤ 0.01.

ARWMC, Age Related White Matter Changes; BMS, Burning Mouth Syndrome; NRS, Numeric Rating Scale; SF-MPQ, Short-Form McGill Pain Questionnaire; HAM-A, Hamilton Anxiety; HAM-D, Hamilton Depression; PSQI, Pittsburgh Sleep Quality Index; ESS, Epworth Sleepiness Scale; PT, prothrombin time; INR, international normalized ratio; aPTT, partial thromboplastin time; AT III, antithrombin 3; Anti-B2GPI, anti-B2-glycoprotein 1; ACA, anticardiolipin antibodies; LAs, lupus anticoagulants; CRP, C reactive protein.

Therefore, the most important predictors of the total ARWMCs score were age, education, amount of drug intake, systemic comorbidities, essential hypertension and hypercholesterolemia.

A detailed linear correlation analysis between the sub-scores of the ARWMCs and the above identified significant predictors correlated with the total ARWMCs score is shown in Table 6. Among the quantitative predictors identified, only age was found to be strongly and positively correlated with the RF and LF lobe scores and RPO and LPO lobe scores (*p*-values: <0.001^**^). On the other hand, among the qualitative predictors, there was a statistically significant correlation only between essential hypertension and the RF, LF, RPO and LPO lobe scores (*p*-values: 0.003^**^, <0.001^**^, <0.001^**^, <0.001*, respectively).

**TABLE 6:** Linear correlation analysis between the ARWMC sub-scores and statistically significant quantitative/qualitative predictors of the total ARWMC score in the BMS patients.

Quantitative predictors		Right frontal		Left frontal		Right parieto-occipital		Left parieto-occipital		Right temporal		Left temporal		Right basal ganglia		Left basal ganglia		Right infraten -torial		Left infraten -torial		Total score
		ρ (*P*-value)		ρ (*P*-value)		ρ (*P*-value)		ρ (*P*-value)		ρ (*P*-value)		ρ (*P*-value)		ρ (*P*-value)		ρ (*P*-value)		ρ (*P*-value)		ρ (*P*-value)		ρ (*P*-value)
**Age**		0.341 (0.001*)		0.371 (<0.001*)		0.452 (<0.001*)		0.464 (<0.001*)		0.207 (0.039)		0.104 (0.303)		−0.021 (0.835)		0.139 (0.166)		−0.024 (0.81)		0.099 (0.329)		0.447 (<0.001*)
**Educational levels**		−0.152 (0.132)		−0.179 (0.074)		−0.215 (0.032)		−0.251 (0.012)		−0.048 (0.636)		−0.109 (0.279)		0.025 (0.802)		−0.109 (0.279)		0.041 (0.687)		−0.075 (0.457)		−0.24 (0.016)
**Number of drug intake**		0.247 (0.013)		0.251 (0.012)		0.169 (0.093)		0.152 (0.132)		0.029 (0.777)		−0.028 (0.784)		−0.139 (0.169)		−0.057 (0.575)		0.008 (0.935)		0.1 (0.32)		0.223 (0.026)
**Number of systemic comorbidities**		0.236 (0.018)		0.257 (0.01)		0.231 (0.021)		0.221 (0.027*)		−0.008 (0.933)		0.018 (0.858)		−0.031 (0.762)		0.032 (0.749)		−0.066 (0.514)		0.044 (0.664)		0.266 (0.007)

**Quantitative predictors**	**Right frontal**		**Left frontal**		**Right parieto-occipital**		**Left parieto-occipital**		**Right temporal**		**Left temporal**		**Right basal ganglia**		**Left basal ganglia**		**Right infraten-torial**		**Left infraten-torial**		**Total score**	
	**Median** **Q1:Q3**	***p*-** **value**	**Median** **Q1:Q3**	***p*-** **value**	**Median** **Q1:Q3**	***p*-** **value**	**Median** **Q1:Q3**	***p*-** **value**	**Median** **Q1:Q3**	***p*-** **value**	**Median** **Q1:Q3**	***p*** **-value**	**Median** **Q1:Q3**	***p*-** **value**	**Median** **Q1:Q3**	***p*-** **value**	**Median** **Q1:Q3**	***p*-** **value**	**Median** **Q1:Q3**	***P*-value**	**Median** **Q1:Q3**	***p*-** **value**

**Employment status** *Employed* *Not employed*	1 [0–1] 1 [0.5–1]	0.042	1 [0–1] 1 [0–1]	0.077	0 [0–0] 0 [0–1]	0.009	0 [0–0] 0 [0–1]	0.025	0 [0–0] 0 [0–0]	0.462	0 [0–0] 0 [0–0]	0.766	0 [0–0] 0 [0–0]	0.532	0 [0–0] 0 [0–0]	0.670	0 [0–0] 0 [0–0]	0.438	0 [0–0] 0 [0–0]	0.438	2 [0–2] 2 [1–4.5]	0.014
**Essential hypertension** *Yes* *No*	1 [1–1] 1 [0–1]	0.003*	1 [1–1] 0 [0–1]	<0.001*	1 [0–1] 0 [0–0]	<0.001*	1 [0–1] 0 [0–0]	<0.001*	0 [0–0] 0 [0–0]	0.676	0 [0–0] 0 [0–0]	0.593	0 [0–0] 0 [0–0]	0.829	0 [0–0] 0 [0–0]	0.406	0 [0–0] 0 [0–0]	0.388	0 [0–0] 0 [0–0]	0.768	3.5 [2–5] 1.5 [0–2]	<0.001*
**Hypercholes** **-terolemia** *Yes* *No*	1 [1–1] 1 [0–1]	0.149	1 [0–1] 1 [0–1]	0.549	1 [0–1] 0 [0–1]	0.011	1 [0–1] 0 [0–1]	0.046	0 [0–0] 0 [0–0]	0.532	0 [0–0] 0 [0–0]	0.355	0 [0–0] 0 [0–0]	0.127	0 [0–0] 0 [0–0]	0.288	0 [0–0] 0 [0–0]	0.475	0 [0–0] 0 [0–0]	0.059	3 [2–5] 2 [0–4]	0.039

The significance difference between correlation coefficients was measured using the Bonferroni correction. The test is significant with a *P*-value < 0.005*.

A significant difference between medians was measured using the Mann–Whitney *U* test with the Bonferroni correction. The test is significant with a *P*-value < 0.005.

ARWMC, Age Related White Matter Changes BMS, Burning Mouth Syndrome.

## Discussion

Burning Mouth Syndrome is a type of complex chronic neuropathic orofacial pain disorder, frequently associated with emotional and cognitive impairment (de Souza et al., 2012; Canfora et al., 2021). It is known that changes involving the brain structure and networks, typical of central neuropathies, play a key role in the aetiopathogenesis of disease (Khan et al., 2014; Wada et al., 2017; Kurokawa et al., 2021). Currently, any BMS diagnosis is exclusively based on clinical features and laboratory findings. However, recently, Canfora et al. (2021) have found in a sample of 40 BMS patients a high prevalence of WMHs, mainly in the temporal lobe, highlighting a possible correlation between the localization of the WMHs and pain perception. The increment in the number of patients recruited showed that also the frontal and parieto-occipital lobes were more involved in BMS patients, while in the basal ganglia area there were not any significant differences. These findings suggest that it could be useful to request a MRI of the brain in patients with BMS (Khan et al., 2014; Wada et al., 2017; Canfora et al., 2021; Kurokawa et al., 2021).

The present study provides further data on the presence of WMHs in a wide sample of patients with BMS compared with a control group of healthy subjects matched for age and gender. This is the first study which investigates and quantifies the WMHs in BMS patients, also analyzing any potential predictors. WMHs have been identified in 50% of patients with BMS and in 26% of healthy subjects. The WMH localization shows a higher prevalence of WMHs in the frontal, parieto-occipital and temporal areas of the brain in BMS patients (Figure 3) in comparison with the control group. On the contrary, the infratentorial and basal ganglia regions are very rarely affected by WMHs in either group. This difference is predominant in BMS patients aged under 65 and over 75, suggesting that the onset and extension of WMHs could occur earlier in patients with BMS. This may affect the health of the brain and produce a premature aging of the brain (Xiong et al., 2010; Debette et al., 2019), which could explain the higher prevalence of WMHs in patients older than 75 compared with the healthy subjects. It is known that chronic pain could stimulate a premature aging of the brain (Higgins et al., 2018), causing damage in its structure and functionalities, involving a decrease in the brain neuroplasticity (Innes and Sambamoorthi, 2020), notwithstanding the individual’s chronological age (Cao et al., 2019). In this context, the presence of WMHs may be an additional biomarker of brain frailty, promoting a progressive aging and making the brain less prone to respond continually to environmental stimuli, reducing neuroplasticity which in turn may reciprocally aggravate the pain perception (Galluzzi et al., 2008; De Marco et al., 2017; Frizzell et al., 2020).

Several studies have shown that WMHs are progressive and their severity correlates with cognitive decline and mortality, common findings in relation to AD and VaD (Xiong et al., 2010; Griffanti et al., 2018; Debette et al., 2019; Bauer et al., 2021). Therefore, any interception in the earliest stages could be an opportunity to prevent, or even reverse, this brain damage, delaying the development of dementia and reducing the risk of stroke and mortality (Galluzzi et al., 2008; Maillard et al., 2014; Alosco et al., 2018). In addition, a higher prevalence of WMHs in BMS patients, predominantly in those older than 75, could predict a rapid global functional decline, occurring in approximately 3 years (Moon et al., 2017). The last stage of this decline is the transition to disability, as suggested in the study of Leukoaraiosis and the disability study group (LADIS) (The Ladis Study Group et al., 2011).

Given the clinical relevance and the prognostic value of WMHs (Maillard et al., 2014; Moon et al., 2017), quantifying and monitoring over the time the progression of WMHs in BMS patients may be clinically crucial in terms of a comprehensive management of the patient, and could add new perspectives in this emerging area of research (Higgins et al., 2018).

Structural imaging based on MRI of the brain is a mainstay in the clinical assessment and differential diagnosis of patients with suspected neurological diseases such as AD and VaD (Moon et al., 2017; Kim et al., 2022). It can also be effective to exclude neurodegenerative disorders in younger patients with BMS (Zhang and Meng, 2015; Di Stefano et al., 2019). In particular, simple scales, such as the ARWMCs and Fazekas (Gouw et al., 2008), are an applicable method to quantify WMHs in different brain areas, offering an opportunity for early diagnosis and potential interventions in vascular diseases which affect the brain. MRI of the brain is a non-invasive high resolution imaging technique, free of ionizing radiation and relatively low-priced (Yousaf et al., 2018). However, it is not possible to perform this investigation with claustrophobic and bariatric patients and with patients supporting any cardiac implantable electronic device (Ghadimi and Sapra, 2022).

Despite such findings, currently, there is no evidence of the request for MRI in patients with COFP. Structural MRI is a common screening tool used by clinicians in the differential diagnosis of several types of COFP in order to exclude structural lesions, such as intracranial tumors and cysts, or any vascular compression of the trigeminal nerve (Ohba et al., 2014; Devine et al., 2019). In a recent study of Devine et al. (2019) on 125 patients with different types of COFP, except BMS, the authors found no alteration in the MRI scan in 51.2% of patients. Instead, 48.8% of patients showed an intracranial pathology, respectively, trigeminal neurovascular contact (22.4%), cerebral small vessel disease (CSVD) (20%), space-occupying lesions (2.4%), a pineal cyst (1.6%), sinus pathologies (1.6%) and degenerative changes to the cervical spine (0.8%). These percentages are lower compared with the study of Ogütcen-Toller et al. (2004) on a sample of 38 patients affected by trigeminal neuralgia or atypical facial pain in which neurovascular compression of the trigeminal nerve was found in 39.5% and space-occupying lesions in 15.7% of patients. Notwithstanding this, MRI scanning of the brain has never been recommended in the assessment of patients with BMS. However, the findings of this study may represent a starting point for the development of BMS assessment guidelines, also including MRI, in order to evaluate the presence of WMHs not only for structural lesions of the brain.

As reported by the study of the Devine (Devine et al., 2019), CSVD was found in 20% of patients with COFP and the neuroimaging markers of CSVD are primarily WMHs, in addition to lacunes, small subcortical infarcts, perivascular spaces, cerebral microbleeds and brain atrophy. In accordance with this research, the results of the present study suggest that the high prevalence of WMHs in BMS patients may also be a manifestation of CSVD, resulting, over time, in VaD or AD (Brown et al., 2018; Tariq et al., 2018). Indeed, the progressive cognitive decline frequently involves a double etiology both neurodegenerative and vascular (Korczyn et al., 2012; Stephen et al., 2021), suggesting the therapeutic relevance of treating vascular risk factors (Korczyn et al., 2012), such as essential hypertension and hypercholesterolemia. Moreover, several studies have suggested that high blood pressure, appearing earlier in life, is associated with an increased quantity of WMHs, evident only five to 20 years later (Beauchet et al., 2013; Wartolowska and Webb, 2021). Indeed, antihypertensive treatment has a protective role in reducing its progression (Messerli et al., 2011). In this study, essential hypertension and hypercholesterolemia have been found respectively in 58% and in 46% of patients with BMS and these systemic comorbidities, after age, are the most important predictors of the ARWMCs total score as suggested by the correlation analysis. Specifically, only hypertension correlated with the sub-scores of ARWMC in frontal and parieto-occipital lobes. Moreover, in this study HHCys was found in 73% of BMS patients. The prevalence of HHCys in our sample is higher compared with the study of Chiang et al. (2019) al where the authors found HHCys in 19.2% of 884 patients with BMS. Even if in this study no direct correlation was found between HHCys and WMHs, it is important to consider that recent evidence suggests HHCys as a novel pathogenetic factor in the onset of WMHs (Wright et al., 2005; Ansari et al., 2014; Lee et al., 2019) because it may induce endothelial dysfunction and extracellular matrix proliferation leading to vascular damage (Hsu et al., 2020). In addition, as suggested by the Rotterdam Scan Study (Vermeer et al., 2002), plasma Hcyt levels are not only associated with the overall risk of having a stroke, CSVD and AD, but are also significantly associated with an increased risk of cerebral WMH progression (Vermeer et al., 2002; Dufouil et al., 2003a), after adjusting for other confounding factors. Therefore, it is crucial to control the level of HCys in order to reduce the risk of WMHs progression and consequently to prevent AD, VaD and dementia (Wright et al., 2005).

In addition, the higher prevalence of WMHs in the frontal, parieto-occipital and temporal areas of the brain may be related to the lenticulostriate branches of the anterior part of the Circle of Willis and the middle cerebral arteries, which are the most affected vascular regions hit by arteriosclerosis (Denswil et al., 2016).

Surprisingly, despite the fact that the majority of BMS patients report severe pain, in terms of the intensity and quality of pain in association with a higher prevalence of anxiety, depression and sleep disturbances, no correlation was found between the ARWMCs total score and pain, mood disorders and disease duration, suggesting a different etiopathogenetic model in the development of WMHs in BMS patients.

However, WMHs have been considered as one of the major contributors to the vascular hypothesis of late-life depression and cognitive decline in the elderly (Galluzzi et al., 2008; Moon et al., 2017; Chang et al., 2019). It is proposed that cerebrovascular factors predispose, precipitate or perpetuate geriatric depressive syndromes (Göthe et al., 2012). Protective factors, such as higher education and cognitive reserve improvement, could mitigate the negative association between WMHs and late life depression (Dufouil et al., 2003b; Scarmeas and Stern, 2004). In our study, education is negatively correlated with the total ARWMCs score, suggesting that it has a role in the development of WMHs, further aggravating the psychological profile of BMS patients.

In this scenario, it seems reasonable to consider WMHs as a silent early marker of brain frailty (Siejka et al., 2020). The results of the present study have contributed to the addition of knowledge about their role in the pathophysiology of BMS, which is still not totally clear. The assessment and treatment of patients with BMS continue to be a challenge for clinicians and, in accordance with this study, a correct diagnosis should include, not only the evaluation of pain scales and the psychological profile, but also the analysis of patients’ metabolic profile. Considering our results we could state that the medical history of the patients, which includes their comorbidities and drugs intakes, in association with the cardiovascular risk factors persistence, may have a role in WMH and BMS development. Thereby the treatment of hypertension and hypercholesterolemia along with the therapeutic modulation may both reduce the WMH progression and improve the BMS patients’ prognosis. The high prevalence of WMHs in patients with BMS, taking in account that WMH dynamic progression in brain parenchyma has a rate ranging from 0.1 to 2.2 ml/year (Jiménez-Balado et al., 2022), increasing the risk of developing cognitive decline, dementia and disability, confirms the usefulness of requesting MRI of the brain during the first consultation. This early screening could allow the evaluation of WMHs and the measurement of their progression and/or regression over time, after controlling for vascular risk factors (Debette et al., 2019). Therefore, every patient should be screened for vascular risk factors by routine laboratory tests, including a complete metabolic panel, lipid profile, and HCys in the first consultation and during the follow-up. Indeed, the monitoring of blood pressure, dietary modifications and weight control, a low level of HCys and the improvement of physical and cognitive activities all contribute to decrease the progression of WMHs and simultaneously enhance the cognitive reserve (Ferri et al., 2020; Stephen et al., 2021; Chowdhary et al., 2022).

## Conclusion

These results support the hypothesis that patients with BMS show a high prevalence of WMHs, mainly in the frontal, parieto-occipital and temporal areas of the brain. WMHs are correlated with age and naturally increase in older age. However, in BMS patients, WMHs may occur earlier contributing to the premature aging of the brain. An aging brain, in turn, may have an impact not only on worsening the pain perception and aggravating the mood disorder but also as a predictive factor for the patient in terms of indicating the development of a neurodegenerative disease, such as AD and VaD. Accordingly, clinicians should consider including the analysis of cardiovascular risk factors and the performance of MRI of the brain as adjunctive tools in the assessment of BMS patients. Moreover, the treatment of patients with BMS should be focused not only on pain modulation and the improvement of mood disorders, but also on the reversing of brain alterations such as WMHs, through the monitoring and treatment of modifiable cardiovascular risk factors and the promotion of correct lifestyle behaviors.

## Limitations

The results of this study should be interpreted in the context of the following limitations. First, the study does not include any information about the sociodemographic characteristics, comorbidities, drug intake and blood biomarkers of the control group. Secondly, the study sample may reflect a selection bias. The prevalence of WMHs in BMS patients may be different dependent on the institution because it can vary in accordance with different brain MRI examination criteria and other factors. Thirdly, it is not possible to define, on account of this being a cross-sectional study, a causal effect between pain and WMHs. Finally, we did not explore other factors potentially causing differences in ARWMC scores and their distribution in patients and controls with different ages. Future prospective studies are needed to confirm our conclusions.

## Data availability statement

The raw data supporting the conclusions of this article will be made available by the authors, without undue reservation.

## Ethics statement

The studies involving human participants were reviewed and approved by Ethical Committee of the University (Approval Number: 251/19- date of approval 20th February 2019). The patients/participants provided their written informed consent to participate in this study.

## Author contributions

All authors listed have made a substantial, direct, and intellectual contribution to the work, and approved it for publication.
