# Supplementary material for: White matter hyperintensities in Burning Mouth Syndrome assessed according to the Age-Related White Matter Changes scale
Source: Front Aging Neurosci. 2022 Sep 1;14:923720. doi: 10.3389/fnagi.2022.923720 (PMC9475000; doi:10.3389/fnagi.2022.923720)
Supplement: Supplementary file 1 [file Data_Sheet_1.docx]

**Supplementary 1**

**Results**

A total of 100 BMS patients were included in the present study. The **Table 1** showed the sociodemographic profiles, risk factors, systemic diseases, drug consumption and biochemical blood biomarkers. It revealed that female patients were predominant over males accounting for 76% of the total sample; the mean of the age was 65.34 ± 8.14 and of the education duration was 9.09 ± 4.44 years. 82% of the BMS patients were married and 46% were unemployed. The majority of the BMS patients (76%) reported that they had never smoked and 85% did not consume alcohol. With respect to the biochemical biomarkers, the medians and IQR ranges of homocysteine were 13.95 [10.95-16]. Specifically, 73% of the patients showed HHCys [59 females (77.63%) and 14 males (58.33%)]. In addition, only 4% of the BMS patients engaged in any regular physical activity. **Figure 2** shows the systemic comorbidity and drug intake in BMS patients: essential hypertension (58%), hypercholesterolemia (46%), gastroesophageal reflux disease (18%) and hypothyroidism (15%) were the most common medical comorbidities recorded. Consequently, statins (34%), proton pump inhibitors (28%), antiplatelets (25%), ACE-inhibitors (23%), beta-blockers (18%), angiotensin receptor blockers (17%) and levothyroxine sodium (11%) were the most frequently prescribed drugs.

**Table 2** shows the prevalence of the oral symptoms, the location, timing and pattern of the pain, the number of specialists consulted and typology of referrals, the analysis of the intensity and quality of pain, and the analysis of the psychological profile of the patients. Besides the burning sensation, which is the distinctive oral symptom in BMS, the other additional oral symptoms affecting the majority of the BMS patients were xerostomia (56%), dysgeusia (44%), change in tongue morphology (44%) and globus pharyngeus (37%). The burning sensation was the worst symptom reported by 76% of the patients, affecting predominantly the tongue (94%), the anterior palate (60%) the gums (55%) and the buccal mucosa (53%). The symptoms presented with different patterns over the day, being continuous in nature in almost 69% of cases.

The BMS patients reported an onset of the symptoms an average of 29 ± 47.67 months prior to the diagnosis, and reported having consulted an average of 2.6 specialists, mostly dentists (90%) and general physicians (53%), followed by maxillofacial surgeons (17%), gastroenterologists (17%) and otolaryngologists (15%). Many of the patients (66%) could not find any cause triggering the oral symptoms, while 22% and 12% attributed the development of the disease to dental treatments and to stressful life events, respectively. Overall, the BMS patients complained of severe pain, both in terms of the intensity and quality of pain, shown by a VAS median score of 10 (IQR: 9.75-10) and by a SF-MPQ median score of 9 (IQR: 5.75-13). The participants also manifested symptoms of anxiety and depression with median scores in both the HAM-A and HAM-D of 18 (IQR: 15-24; and 14-21, respectively), almost 30% having a previous history of mood disorders. Specifically, 44% of the BMS patients suffered from mild anxiety (HAM-A score <17), 49% from moderate anxiety (HAM-A score 18-24) and 7% from severe anxiety (HAM-A score 25-30). Instead, depression was mild in 46% of the patients (HAM-D score 7-17), moderate in 42% (HAM-D 18-24) and severe in 12% (HAM-D score > 24). In addition to an impairment of the psychological profile, 86% of the BMS patients presented a poor quality of sleep (PSQI: median; IQR: 8[8-10]) with a short sleep duration (median; IQR 5: [5-6], while only 5% of the patients manifested mild to moderate daytime sleepiness (ESS score 11-17). Interestingly, the 74% of the patients complained of the onset of insomnia a median of 4 years prior to the diagnosis of BMS (IQR: 2-6 years).
